# A Deep Learning-Based Approach to Video-Based Eye Tracking for Human Psychophysics

**DOI:** 10.3389/fnhum.2021.685830

**Published:** 2021-07-21

**Authors:** Niklas Zdarsky, Stefan Treue, Moein Esghaei

**Affiliations:** ^1^Cognitive Neuroscience Lab, German Primate Center – Leibniz Institute for Primate Research, Göttingen, Germany; ^2^Faculty of Biology and Psychology, University of Göttingen, Göttingen, Germany; ^3^Bernstein Center for Computational Neuroscience, Göttingen, Germany; ^4^Leibniz-ScienceCampus Primate Cognition, Göttingen, Germany

**Keywords:** deep learning, eye tracking, gaze tracking, artificial intelligence, DeepLabCut, human psychophysics

## Abstract

Real-time gaze tracking provides crucial input to psychophysics studies and neuromarketing applications. Many of the modern eye-tracking solutions are expensive mainly due to the high-end processing hardware specialized for processing infrared-camera pictures. Here, we introduce a deep learning-based approach which uses the video frames of low-cost web cameras. Using DeepLabCut (DLC), an open-source toolbox for extracting points of interest from videos, we obtained facial landmarks critical to gaze location and estimated the point of gaze on a computer screen via a shallow neural network. Tested for three extreme poses, this architecture reached a median error of about one degree of visual angle. Our results contribute to the growing field of deep-learning approaches to eye-tracking, laying the foundation for further investigation by researchers in psychophysics or neuromarketing.

## Introduction

Tracking the point of gaze as a window to the internal state of the human mind is a key requirement in cognitive tasks, where it is important to control the attention of human subjects. This application has been the focus of classic studies (Yarbus, 1967), and more recently, the approach has been used not only as a clinical tool to detect neurological and neuropsychiatric disorders by studying the patients’ gaze patterns (Adhikari and Stark, 2017), but also has been shown to be useful in every-day applications, such as analyzing the trustworthiness of phishing emails (McAlaney and Hills, 2020). While there are different existing technologies to eye tracking on the market, an affordable and practical technology is still missing, limiting the use of this technique to a broader audience.

There are two major approaches to video camera-based eye-tracking: model-based and appearance-based. Model-based approaches calculate the point of gaze using a 3D model of the eye and the reflected infrared patterns on the cornea. Guestrin and Eizenman (2006) documented that pose-invariance of gaze estimation is highly dependent on the number of cameras and infrared light sources, suggesting a stronger pose-invariance when using more than a single infrared source (see Eyelink 1000 system-SR Research, Mississauga, ON, Canada as an example of a pose-invariant model-based eye-tracking system with an array of infrared LEDs). Chen et al. (2008) eliminated the errors induced by the 3D model, using a novel mathematical approach. The appearance-based approach uses only the video camera’s image data. Here, machine-learning techniques are often used to map the extracted coordinates of facial landmarks to the respective point of gaze (Lu et al., 2017). This is a straightforward problem when the subject’s head is stationary, leading to a simple transformation of facial landmarks’ relative positions to the point of gaze. However, when under various head poses, this transformation would differ as a function of the specific head pose. Here, machine learning-based approaches could estimate such complex relations between the position of facial landmarks and the point of gaze (Ranjan et al., 2018). But note that proposed open-source machine learning approaches have not yielded sufficient accuracy [>3 degrees visual angle (dva)] appropriate for psychophysics experiments (Zhang et al., 2017; Lemley et al., 2018).

Hence, we propose a new appearance-based eye-tracking method, using a simple webcam while maintaining the advantages of previous approaches, helping to make eye tracking available to a broader audience: it reduces the high acquisition costs of eye-tracking systems [high-end devices cost up to 40,000€ (Rakhmatulin, 2020)] and could also be implemented in space-restricted use cases (like mobile environments) where multiple cameras and infrared light sources are not available.

We introduce a two-stage pipeline; Stage 1: We extract the facial landmarks critical to the estimation of gaze from the raw video frames. For this, we utilize the DeepLabCut (DLC, version 2.1.6.2)^1^ toolbox, a deep learning-based object-tracking framework widely used for motion tracking in animals, based on hand-labeled landmarks (Mathis et al., 2018; Nath et al., 2019). Stage 2: GazeNet, a shallow, self-developed feed-forward neural network, maps the extracted landmark coordinates to the gaze position. This pipeline yields sufficient accuracy while it is not restricted to a single pose. Our results indicate that this pipeline is a proof of concept for a low-cost, multi-pose robust eye-tracking system.

## Materials and Methods

### Recording

All data were recorded with a Logitech C922 Pro Stream video camera (Logitech Europe S.A., Lausanne, Switzerland) at a frame rate of 30 fps. For the tracking task, a 27-inch (595 mm × 335 mm) 1440 p (2560 × 1440) iMac (Apple, Cupertino, CA, United States) was used. All computations were run on a Mac mini, 3.2 GHz i7, 16 GB RAM (Apple), no dedicated graphics card.

Each recording session consisted of a small solid circle traversing the screen horizontally, followed by a vertical increment toward the bottom of the screen. This is repeated in a continuous fashion until the vertical end is reached. This behavioral paradigm lasted approximately 1 min (2.5 min for the two additional subjects, because of more horizontal lines and a slower movement of the circle). The subjects were asked to follow the moving dot across the screen, while seated at a distance of approximately 50 cm to the screen (eye distance to screen).

To allow the subject to track the circle more easily, it slowed down at direction changes. A speed-up during the long horizontal paths kept the duration of the session short.

Because of the slow-down at the edges, more data points were sampled toward the sides of the screen. To remove this bias, the screen was divided into square regions with the side length of 530 px. This resulted in 15 squares across the screen surface, with the number of samples ranging between [28, 101] (std = 26.8) ([42, 92] (std = 15.1) and [43, 96] (std = 16.2) for the two additional subjects). We next took the smallest number of points across the regions and randomly selected the same number of points from all regions.

All the extracted data of three different poses were combined into one dataset, which had a size of 3,569 frames. Additionally, frames were removed from the dataset where the likelihood score reported by DLC for at least one landmark was below 0.7 (2.7% of all frames). This way, corrupted frames, e.g., frames showing closed eyes, were omitted from further analyses.

### DeepLabCut

Frames with a low likelihood score were excluded using the DLC network refinement function “extract_outlier_frames.”

The manual estimation of landmark positions was adjusted using the function “refine_labels.”

For training the model using the new dataset, we first called the setup function “create_training_dataset” and next, the function “train_network.”

### GazeNet

GazeNet is a shallow feed-forward artificial neural network, implemented using PyTorch. It consists of 14 input neurons (the x and y coordinates of the seven extracted facial landmarks). The input layer was fully connected to the hidden layer (*N* = 200 neurons; assigned heuristically by balancing between error rate and convergence speed), implemented by a sigmoid activation function. The hidden layer was also fully connected to the output layer, consisting of two output neurons for the x and y coordinate of the estimated point of gaze. Furthermore, GazeNet uses the Stochastic Gradient Descent (SGD) method to accelerate the process of finding the minimum of the loss function, with a learning rate and momentum of 0.03 and 0.6, respectively, also selected manually based on observing the resulting error rate/convergence speed for the training and validation data. Each 2,000-learning epochs, the learning rate is halved by a scheduler to efficiently account for the increasingly smaller steps toward an optimal solution. The number of frames examined before adjusting GazeNet’s parameters in each epoch (batch size) was set to four.

The chosen error function is a derivation of the mean squared error (L2 loss):

$${ℒ}_{2}=\frac{\sum_{i=1}^{n}{\left(t_{i}-{\hat{t}}_{\text{i}}\right)}^{2}}{n}$$

where *n*, *t* and $\hat{t}$ denote the sample size, ground truth and the estimated gaze location, respectively.

For the data used in this work, containing x and y coordinates, the difference of the ground truth and the estimation is calculated as follows:

$$t-\hat{t}=\left(t_{x},t_{y}\right)-\left({\hat{t}}_{x},{\hat{t}}_{y}\right)=\left(\Delta x,\Delta y\right)$$

The loss function (in pixels) is calculated using the following formula:

$$E_{px}=\frac{\sum_{i=1}^{n}\sqrt{\Delta x_{i}^{2}+\Delta y_{i}^{2}}}{n}$$

Next, we used the following formula to calculate the error in degrees of visual angle from the error in pixels:

$$E_{dva}=\arctan\left(\frac{E_{px}}{d_{mm}}\cdot \frac{sw_{mm}}{sw_{px}}\right)\cdot \frac{180}{\pi }$$

*E*_*px*_, *d*_*mm*_, *sw*_*mm*_, *sw*_*px*_ denote the error in pixels, distance between the subject’s eyes and the screen in mm, the screen width in mm and the screen width in pixels, respectively.

## Results

**FIGURE 1:**
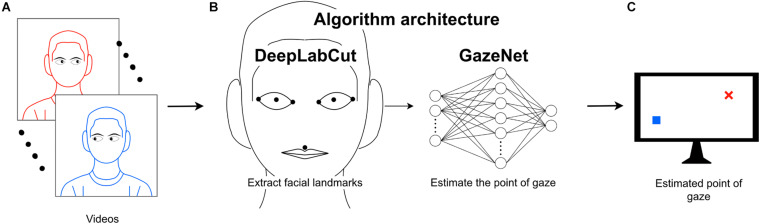
Outline of the algorithm architecture. **(A)** Video frames of the subjects captured while they are tracking a small circle on the screen **(B)** DeepLabCut (DLC) is capable of tracking visual entities and is used to track the specific facial landmarks from the input video frames. An additional artificial neural network (GazeNet) is provided with the landmark positions together with the respective coordinates of the moving stimulus presented on the screen. **(C)** GazeNet estimates the coordinates of the target location on the screen based on the extracted facial landmarks.

**FIGURE 2:**
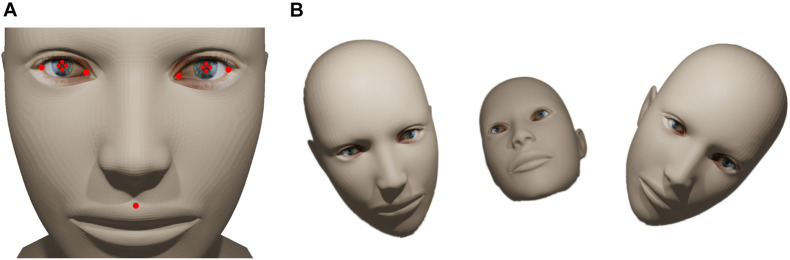
Facial landmarks and pose variants. **(A)** The red dots denote the landmarks tracked by DLC. **(B)** Schematic of the three different poses used to train and evaluate GazeNet (human head model design obtained from Thingiverse.com, #758647, designed by lehthanis, license: CC BY-SA 3.0, modified neck, added textures).

To estimate the location of gaze using videos of the face, we recorded video frames of a subject while the subject was tracking a small circle moving on a maze traversing the whole surface of the monitor. Initially, 50 (1.4% of all recorded) frames were randomly chosen from the recordings and the following facial landmarks were annotated manually: the two lateral corners, the center of the pupil, the two medial corners and the central point between the upper lip and the philtrum (see Figures 1A–C, 2A). These landmarks were chosen to capture all important data from the eyes and sufficient data of the head pose. The annotation was performed using DLC’s graphical user interface by the experimenter. For all steps concerning GazeNet, the center of the pupil was estimated by averaging the Cartesian location of the four corners of each pupil. The subject carried out the task with three different head poses to ensure independence of the measurements from the pose (Figure 2B). After the selected frames were manually annotated (which took around 30 min), the data were used to train DLC to enable tracking the landmarks (see Methods for details on which subroutines of DLC were used for this).

The tracking accuracy was further improved by extracting a set of 50 frames with a low likelihood^2^. The estimated landmark positions in these frames are then adjusted manually, so that faulty-labeled facial features are in their respective correct position afterward. Next, the improved data are merged into a new dataset. Finally, this dataset is used to further train the current DLC model. The overall training time for DLC lasted about 35 h and the extraction of landmarks’ coordinates by the trained DLC from the frames lasted around 40 min. Next, these coordinates were homogeneously normalized by 600 (camera frames were as large as 500 × 600 pixels). This ensures that while the relative point positions do not change, all coordinates vary between 0 and 1.

The resulting dataset (consisting of only landmark coordinates and corresponding position of the circle on the screen) is then split into three sets: a training set (50%), validation set (25%), and testing set (25%). The training and validation sets are used to train GazeNet.

The network undergoes 15,000 iterations (epochs) on the training set, which lasted about 35 min. The network parameters corresponding to the iteration with the highest accuracy (calculated at a resolution of 10 iterations) over the validation set are saved and used in the upcoming steps.

**FIGURE 3:**
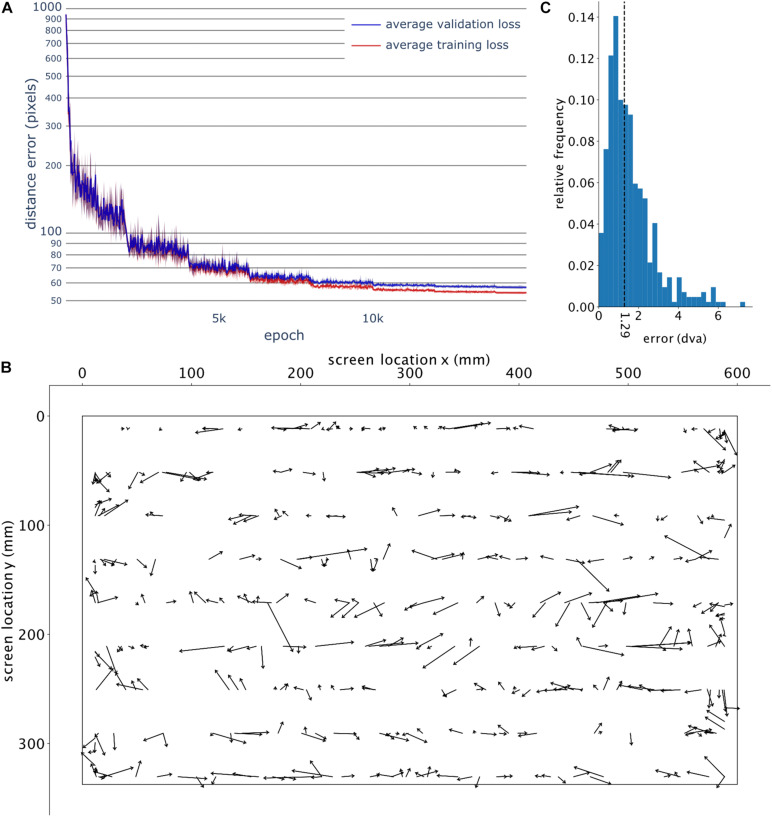
GazeNet’s training course and visualization of error for the test data. **(A)** Course of loss value for the training and validation data, measured by the mean Euclidean distance between the ground truth and the respective estimated location, across the training iterations (epochs). Error bars indicate the standard deviation across 5 distinct training instances. **(B)** The deviation of the estimated points (computed by GazeNet) from the actual location of gaze (for test data). The arrows point from the ground truth toward the estimated location. The outer borders show the boundaries of the monitor. **(C)** Encountered relative frequencies of the estimation error’s magnitude (*n* = 30 bins). The vertical dashed line depicts the median.

GazeNet was trained five times; each time, we selected a random set of frames for training, validation and testing. Figure 3A shows the training and validation loss based on the five different runs for up to 15,000 iterations, to the point where the training loss and validation loss reached a plateau. The high similarity of the accuracy across different runs suggests an independence of the training on the selected frames and the initial parameters of the training algorithm.

The resulting estimations of a single run of the trained GazeNet on the test set are shown in Figure 3B (lasting less than 1 s for all frames). For every sample, an arrow was drawn from the ground truth target location toward the estimated location. Figure 3C shows the histogram of the encountered error magnitudes representing their relative frequencies (median = 1.29 dva). The histogram of error magnitudes for each pose is presented in the Supplementary Figure 1.

**FIGURE 4:**
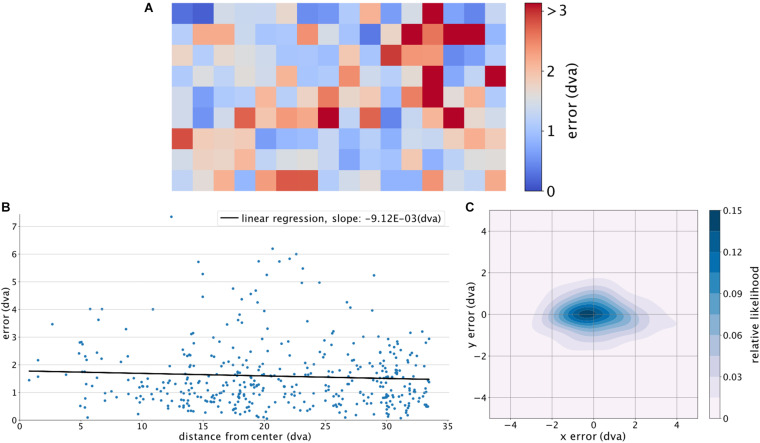
Location-wise analysis of GazeNet’s performance. **(A)** Magnitude of error based on the ground truth locations on the screen, calculated for different regions of the screen. The color bar was limited to the value of the 95% upper bound of the encountered average errors. **(B)** Fitted linear regression on the distance from the center of the screen versus error magnitude. **(C)** Distribution of the estimated point of gaze relative to the actual location of gaze. Position [0,0] indicates the gaze location.

**FIGURE 5:**
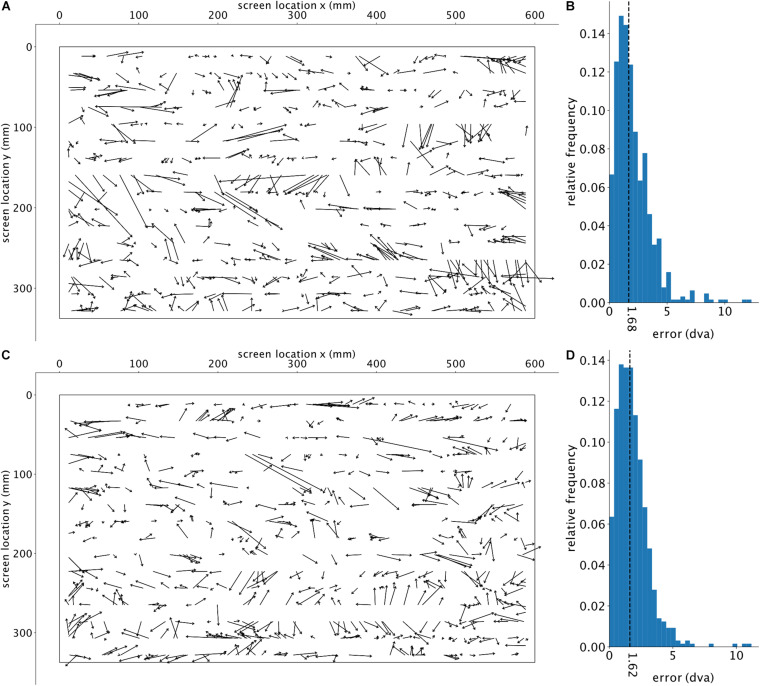
Visualization of test results for two additional subjects. The top row and bottom row each show one subject’s results. **(A,C)** The deviation of the estimated gaze position (computed by GazeNet) from the actual gaze position. The arrows point from the ground truth location toward the estimated position. The outer borders represent the boundaries of the monitor. **(B,D)** Histograms of the relative frequencies of the estimations error’s magnitude, in degrees visual angle (dva). The vertical dashed lines depict the median error. The two subjects’ data included 4068 and 4973 frames (top and bottom row panels, respectively), of which 3.3 and 2.3% were removed due to the DLC’s likelihood threshold.

To further analyze the encountered errors, we divided the screen into 144 equally sized, square regions (of an edge length 160 pixels), for each of which the average error was calculated for the corresponding target points (Figure 4A). The average of the adjacent regions was used to calculate the error for regions with no target points (only non-empty neighboring regions were considered). A fitted linear regression across all the errors relative to their distance (Euclidean) from the center (eccentricity) of the screen shows no significant relationship (*p*-value of 0.208) (Figure 4B), indicating that the estimation accuracy was not systematically dependent on the eccentricity of the target. Using a Gaussian kernel density estimation with a sample grid resolution of 400 × 400 and a granularity of 12 levels to calculate the probability density function, Figure 4C shows a larger error along the horizontal axis. The estimated location is also slightly shifted toward the left side of the target location. To check if our trained algorithm would help detecting new subjects’ gaze locations, we further used the already-trained DLC on two new subjects performing a slightly different continuous gazing task and reached comparable estimation results (see Figure 5). This suggests that with a sufficiently generalized model of DLC, no further training for new subjects will be required.

Table 1 shows where our setup stands compared to other approaches that are appropriate for psychophysics studies in terms of the size of computer screen, processing power, the distance of camera to the subject. A commercial device was also included as an example. This comparison shows that our method is pose-invariant and applicable in limited space, while providing a comparable accuracy at a low cost.

**TABLE 1:** Comparison of the previous studies with our proposed approach.

Studies	Error (dva)	Estimated price ($)	Pose-invariance	Tested for online use	# Cameras	Camera model	Analysis approach	Additional drawbacks
Ours (Zdarsky et al.)	1.29	<100	✓	×	1	Logitech C922 Pro Stream video camera	Appearance-based using deep neural networks and a shallow neural network	
Chen et al., 2008	1	400–1000	✓	✓	2	MINTRON MTV-03K9HE	Model-based with noise reduction	Takes considerable space (due to the stereo camera and infrared light sources)
Lemley et al., 2018	3.64	<100	✓	✓	1	Normal webcams	Appearance-based with a convolutional neural network	
Lu et al., 2011	0.62	<100	×	×	1	Not specified	Appearance-based with linear regression	
Tobii TX300 (Tobii, Stockholm, Sweden)	0.4	> 40,000	✓	✓	Not specified	Not specified	Model-based	

## Discussion

Cognitive neurosciences and neuromarketing rely on gaze tracking (Lim, 2018; Zareian et al., 2020; Parto Dezfouli et al., 2021; Veith et al., 2021); however, a proper solution has been missing due to the high number of degrees of freedom involved with the possible combinations of eye and head movements. Here, we showed that by using a recently introduced pose-estimation tool named DLC, the dimensionality of gaze tracking could be reduced considerably to estimate the point of gaze with a shallow artificial neural network. Using the coordination of seven facial landmarks necessary to capture the gaze-relevant information, our system was able to estimate the gaze location with a median accuracy of 1.29 dva.

Although the DLC here was trained on data from one of the subjects, it was able to extract the relevant facial landmarks from the other two subjects. The results for the additional two subjects suggest that our approach’s performance should be robust for other new subjects.

Our dataset here were recorded under a specific experimental setting (lighting, screen-eye distance, room temperature, elimination of distraction). Nevertheless, given DLC’s image augmentation feature which simulates different environmental settings by artificially modifying the low-level visual features of input frames, we expect the algorithm to be robust also against different environmental settings. Furthermore, there are other sources of variability which may affect the stability of our framework’s results; our framework shall therefore be tested using different video cameras of different resolutions, for a higher number of poses and yet a higher number of different environmental settings as simulated by DLC’s pipeline. Ideally, testing the sensitivity to these sources of variability would depend on the specific use case of our approach. For instance, in a mobile web-based marketing application, where customers’ attention to different sections of an e-marketing website is measured, the framework should be tested for stability against a potential continuous change of the subjects’ head position.

We observed that the error along the X-axis is larger than the error along the Y-axis (Figure 4C). This could be due to the horizontal motion of the tracked dot being faster than the vertical speed. This may have given rise to a less accurate pursuit of the dot by the subject on the horizontal axis. Future studies could resolve this issue by either using a modified path for the dot with curved corners, or sequentially presenting a series of dots on a grid of locations to avoid the artifact of speed differences on the accuracy of the gaze-following behavior.

Even though the dataset was only from one subject, our results indicate that this system is comparable in accuracy, but can be built at a fraction of the cost of other systems. Training our architecture using data from a large-scale dataset [like MPIIGaze (Zhang et al., 2019)], with videos of multiple subjects, a multitude of poses under improper lighting conditions, may help reach higher levels of generalizability. This is not only true for DLC, but also for GazeNet, which may become more generalized using data from different subjects and further train the model based on prior training. However, the shallow design might limit the efficiency of the learning process in terms of generalizability. The overall training time as well as the feature extraction time needed for both DLC and GazeNet could be considerably lowered by using a GPU instead of a CPU. For instance, 682 × 540 pixel frames can be processed at around 30 Hz on an NVIDIA GTX 1080 TI GPU, while videos with a frame size of 204 × 162 could be processed at around 85 Hz (Mathis et al., 2018).

DeepLabCut’s accuracy can be increased by using videos with a higher quality as input (using a higher-resolution camera) or by using a larger number of labeled frames. The performance could be further improved by optimizing GazeNet’s parameters (e.g., number of hidden layers and their respective neurons, learning rate and scheduling parameters) using automatic hyperparameter optimization strategies.

## Data Availability Statement

The raw data supporting the conclusions of this article will be made available by the authors, without undue reservation. Scripts are available from: https://github.com/zdarsky/gazenet.

## Ethics Statement

Prior to the testing written informed consent was obtained from the participating subjects. The experiment was not harmful and experimental data have been treated anonymously. The experiment was performed in accordance with the ethical standards laid down by the 1964 Declaration of Helsinki. We followed the relevant guidelines of the Germany Psychological Society (Document: 28.09.2004 DPG: “Revision der auf die Forschung bezogenen ethischen Richtlinien”) and obtained official approval for these experiments by the Ethics Committee responsible at the University of Göttingen.

## Author Contributions

NZ and ME designed the study and wrote the manuscript. NZ recorded the data and performed data analyses. NZ, ST, and ME interpreted the data. ST critically reviewed the manuscript. All authors approved the final version of the manuscript.

## Conflict of Interest

The authors declare that the research was conducted in the absence of any commercial or financial relationships that could be construed as a potential conflict of interest.

## Footnotes

1 Obtained from https://github.com/DeepLabCut/DeepLabCut, accessed on Mar 2, 2020, licensed under GNU Lesser General Public License v3.0.

2 The likelihood score denotes the level of confidence for a tracked landmark, ranging from 0 to 1 (unlikely to very likely, respectively).
