# Supplementary material for: A Deep Learning-Based Approach to Video-Based Eye Tracking for Human Psychophysics
Source: Front Hum Neurosci. 2021 Jul 21;15:685830. doi: 10.3389/fnhum.2021.685830 (PMC8333872; doi:10.3389/fnhum.2021.685830)
Supplement: Supplementary file 1 [file Data_Sheet_1.docx]

**A Deep Learning-Based Approach to Video-Based Eye Tracking for Human Psychophysics**

Niklas Zdarsky^1^, Stefan Treue^1^, Moein Esghaei^1^

^1^ Cognitive Neuroscience Lab, German Primate Center- Leibniz Institute for Primate Research, Goettingen, Germany


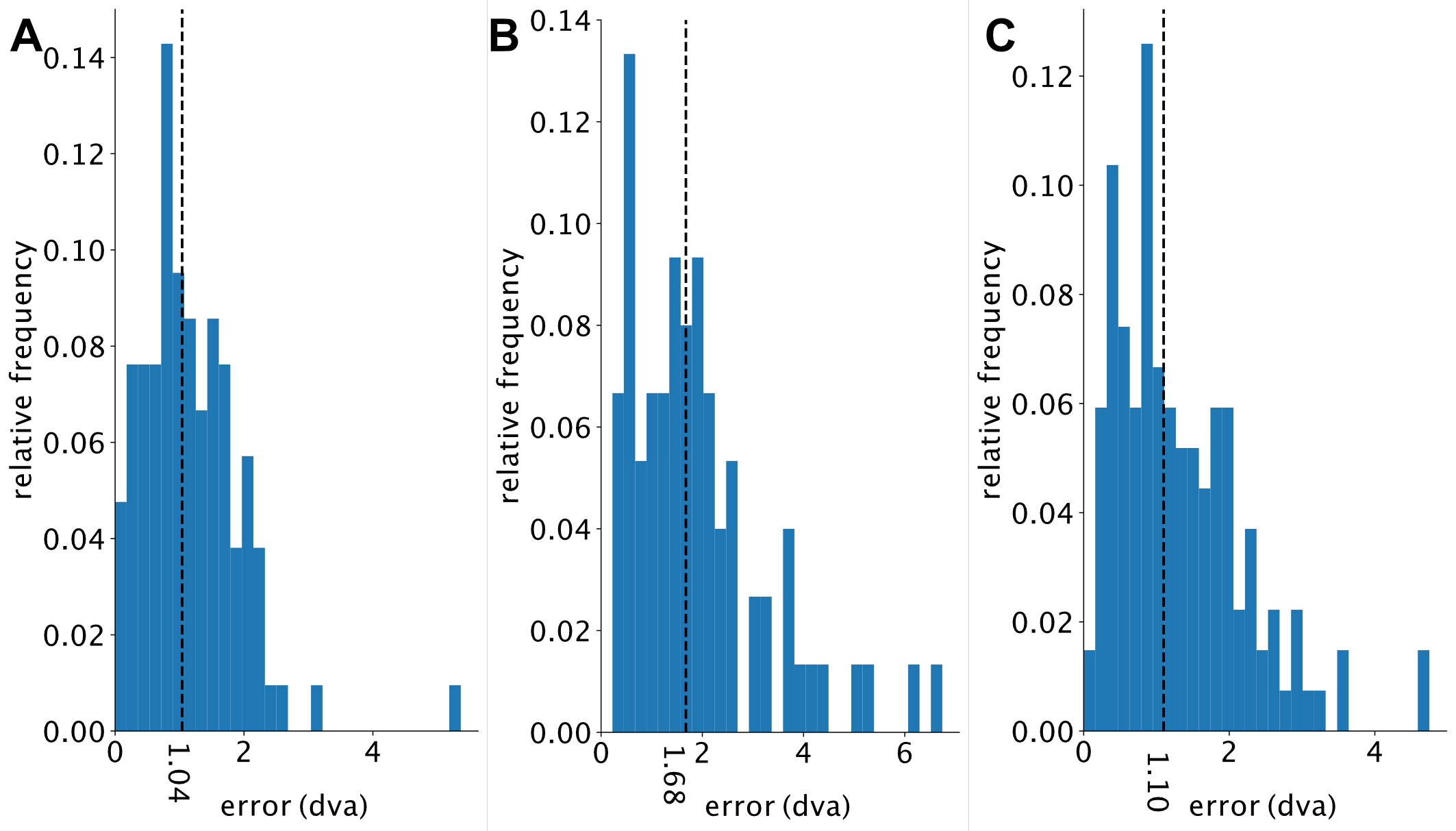


**Supplementary Figure 1:** Histograms of the relative frequencies of the estimations error, in degrees of visual angle (dva). The vertical dashed lines depict the median error. A, B and C refer to the poses shown in Figure 2B (with the same order from left to right).
